# Development of detection method for novel fusion gene using GeneChip exon array

**DOI:** 10.1186/2043-9113-4-3

**Published:** 2014-02-18

**Authors:** Yusaku Wada, Masaaki Matsuura, Minoru Sugawara, Masaru Ushijima, Satoshi Miyata, Koichi Nagasaki, Tetsuo Noda, Yoshio Miki

**Affiliations:** 1 Genome Center, Japanese Foundation for Cancer Research, 3-8-31 Ariake, Koto-ku, Tokyo 135-8550, Japan; 2 Cancer Institute, Japanese Foundation for Cancer Research, 3-8-31 Ariake, Koto-ku, Tokyo 135-8550, Japan; 3 Department of Cardiovascular Medicine, Tohoku University Graduate School of Medicine, 2-1 Seiryo-machi, Aoba-ku, Sendai, Miyagi 980-8575, Japan; 4 Current address: FASMAC Co., Ltd, 3088 Okata, Atsugi City, Kanagawa 243-0041, Japan

**Keywords:** Exon array, Fusion gene, Chromosome rearrangement

## Abstract

**Background:**

Fusion genes have been recognized to play key roles in oncogenesis. Though, many techniques have been developed for genome-wide analysis of fusion genes, a more efficient method is desired.

**Results:**

We introduced a new method of detecting the novel fusion gene by using GeneChip Exon Array that enables exon expression analysis on a whole-genome scale and TAIL-PCR. To screen genes with abnormal exon expression profiles, we developed computational program, and confirmed that the program was able to search the fusion partner gene using Exon Array data of T-cell acute lymphocytic leukemia (T-ALL) cell lines. It was reported that the T-ALL cell lines, ALL-SIL, BE13 and LOUCY, harbored the fusion gene NUP214-ABL1, NUP214-ABL1 and SET-NUP214, respectively. The program extracted the candidate genes with abnormal exon expression profiles: 1 gene in ALL-SIL, 1 gene in BE13, and 2 genes in LOUCY. The known fusion partner gene NUP214 was included in the genes in ALL-SIL and LOUCY. Thus, we applied the proposed program to the detection of fusion partner genes in other tumors. To discover novel fusion genes, we examined 24 breast cancer cell lines and 20 pancreatic cancer cell lines by using the program. As a result, 20 and 23 candidate genes were obtained for the breast and pancreatic cancer cell lines respectively, and seven genes were selected as the final candidate gene based on information of the EST data base, comparison with normal cell samples and visual inspection of Exon expression profile. Finding of fusion partners for the final candidate genes was tried by TAIL-PCR, and three novel fusion genes were identified.

**Conclusions:**

The usefulness of our detection method was confirmed. Using this method for more samples, it is thought that fusion genes can be identified.

## Background

It is well known that cancer is caused by gene abnormalities. There are many types of abnormalities in the genome of cancer cells, including gene fusion because of chromosome rearrangement. The discovery of a characteristic small chromosome, called Philadelphia chromosome, in chronic myeloid leukemia, is the first recurrent chromosome rearrangement to be seen in a human cancer [1]. This rearrangement was eventually identified as a translocation between chromosome 9 and 22 [2], resulting in the fusion of the *BCR* gene on chromosome 22 with the *ABL1* gene on chromosome 9, *BCR-ABL1*[3]. Because many chromosomal abnormalities and fusion genes have been discovered by the development of experimental techniques, it has been shown that such fusion genes and chromosomal abnormalities are causes of cancer. Thus, the importance of chromosomal abnormalities and fusion genes in cancer has been recognized.

It is also known that fusion genes have a key role in oncogenesis in hematological tumors and sarcomas. Since fusion genes are closely related to the clinical and pathological features of tumors, they provide important clues for diagnosis. In addition, fusion genes are regarded as attractive targets of molecular targeted treatments because of their high specificity to tumors.

So far, fusion genes have been found less frequently in common solid cancers, but some reports on prostate [4] and lung carcinomas [5] show that fusion genes contribute significantly to the development of these malignancies. It is predicted that fusion genes have important roles in many other kinds of epithelial tumors [6]. In late years, various fusion genes came to be discovered by many kinds of cancers [7].

Although many technologies are used for the genome-wide screening of fusion genes, there are not yet any versatile methods. Karyotyping requires the availability of fresh, vital cells for short-term culturing to obtain metaphase chromosomes, and it has low resolution. Array comparative genomic hybridization (array CGH) cannot detect fusion genes without genomic copy number change [8]. Recent developments of high-throughput sequencing technologies provide a powerful tool [9-12]. But these technologies are as yet limited by the number of samples that can be analyzed at acceptable cost.

Affymetrix GeneChip Human Exon 1.0 ST Array (Exon Array) is a whole-genome exon expression analysis tool. About 5.5 million probes are being designed on the array, and they compose about 1.4 million probe sets (in principle, the probe set is composed of four probes, and one expression intensity is calculated from one probe set). The expression of almost all exons can be analyzed using the Exon Array, and it enables genome-wide alternative splicing analysis. Each probe set has an ID, and belongs to a transcript cluster that corresponds to a gene. Annotations are given to the probe sets, and are available to the public at Affymetrix NetAffx ( http://www.affymetrix.com/analysis/index.affx). The probe sets are classified into three evidence levels according to the quality of evidence supporting the transcription of the target genomic sequence. The three evidence levels are presented in decreasing order of confidence: "core" (RefSeq and full-length mRNAs), "extended" (ESTs, syntenic rat and mouse mRNAs) and "full" (ab-initio computational predictions). Simultaneously, the probe sets are annotated with hybridization targets that describe cross-hybridization potential. The hybridization targets are shown in decreasing order of uniqueness: "unique", "mixed", and "similar".

In this report, a method to detect abnormal gene structures, including gene fusion, was developed using Exon Array. Using this methodology and TAIL-PCR, novel fusion genes were discovered in breast and pancreatic cancer cell lines. Breast cancer is a heterogeneous disease encompassing a wide variety of pathological features and a range of clinical behavior [13]. These are underpinned at the molecular level by complex components of genetic alterations that affect cellular processes [14]. Therefore, it is possible to contribute for understanding of the heterogeneity and diagnosis with high accuracy by discovering novel fusion genes. Pancreatic cancer is a highly aggressive tumor with no proven curative chemotherapy or radiation therapy, having extremely poor prognosis [15]. The discovery of a fusion gene in pancreatic cancer can lead to molecular target therapy, with the possibility of offering an effective treatment method for pancreatic cancer.

## Methods

### Samples

Twenty-four breast cancer cell lines (AU565, BT474, DU4475, HCC38, HCC70, HCC202, HCC1143, HCC1187, HCC1419, HCC1428, HCC1569, HCC1806, HCC1954, MCF7, MDA-MB-157, MDA-MB-231, MDA-MB-330, MDA-MB-361, MDA-MB-435S, MDAMB-468, SK-BR-3, UACC812, UACC893, ZR-75-1) were obtained from American Type Culture Collection (ATCC), and maintained in under the conditions recommended by the supplier. Twenty pancreatic cancer cell lines (MA005, MA006, PA018, PA022, PA028, PA043, PA051, PA055, PA086, PA090, PA103, PA107, PA109, PA167, PA173, PA182, PA195, PA199, PA202, PA215) were established at Genome Center, Japanese Foundation for Cancer Research (JFCR). Two vials of normal mammary epithelial cells (HMEC), which were donated from different subjects, were obtained from Takara Bio Inc. A non-tumorigenic human breast epithelial cell line (MCF10A) was obtained from ATCC. These were maintained using TaKaRa MEGM BulletKit (Takara Bio Inc, Otsu, Japan) according to the manufacturer’s instructions. A clear cell sarcoma cell line "SarcomaA" was provided by Dr. Nakamura at Cancer Institute, JFCR.

Samples of tumor tissues were obtained from a series of patients with breast or pancreatic cancer who underwent surgery at the JFCR Hospital. All samples were snap-frozen in liquid nitrogen within 1 h after surgery and stored at -80˚C. Before RNA was prepared, laser-captured microdissection (LCM) using a Leica Microsystems AS LMD 600 (Leica Microsystems, Wetzlar, Germany) was performed to ensure that only tumor cells were dissected. LCM was conducted in all tumor samples.

### Open access exon array data

Exon Array CEL files of 17 T-cell acute lymphocytic leukemia (T-ALL) cell lines (ALL-SIL, BE13, CEM, DND41, DU528, JURKAT, KOPTK1, LOUCY, MOLT13, MOLT16, MOLT4, PF382, RPMI8402, SUPT11, SUPT13, SUPT7, TALL1) were obtained from NCBI Gene Expression Omnibus database (Series GSE9342, http://www.ncbi.nlm.nih.gov/geo/query/acc.cgi?acc=GSE9342). It was reported that ALL-SIL, BE13 and LOUCY harbored fusion genes NUP214-ABL1, NUP214-ABL1, and SET-NUP214, respectively [16,17].

### Total RNA extraction and cDNA synthesis

Total RNA was extracted from the cells or the tissues by RNeasy Mini Kit according to the manufacturer’s instructions (Qiagen, Valencia, CA). 1 μg of total RNA was reverse transcribed to synthesize template cDNA by a random primer using the Invitrogen SuperScriptIII FirstStrand Synthesis System(Life Technologies, Carlsbad, California), and 20 μl synthesized cDNA was diluted 500 times with Tris/HCl buffer.

### Exon array experiment

Exon Array data was generated according to the manufacturer’s instructions. Ribosomal RNA was removed from 1 μg of total RNA using Invitrogen RiboMinu Transcriptome Isolation Kit, and amplified cDNA was synthesized using GeneChip WT cDNA Synthesis and Amplification Kit. To make hybridization probes, amplified cDNA was fragmented and biotin-labeled using GeneChip WT Terminal Labeling Kit. The hybridization probes were hybridized to GeneChip Human Exon 1.0 ST Array at 45°C in a hybridization oven at 60 rpm for 16 h, and washed in Fluidics Station 450 using GeneChip Hybridization Wash, and Stain Kit. The array was scanned on GeneChip Scanner 3000 7G. To implement signal summarization, expression intensities for the "core" ProbeSet were calculated using linear normalization and the average-difference method from Affymetrix Power Tools. The median intensity of all arrays was adjusted linearly to 100.

### Fusion gene screening program

The program was developed to detect fusion genes with an exon expression profile similar to that of *EWSR1* and *ATF1* in a clear cell sarcoma cell line, SarcomaA. Details of the program are shown in 1–8

1. To exclude the influence of non-specific hybridization, only probe sets with Hybridization Target "unique" were used.

2. To exclude probe sets that showed extremely low signal intensities in all samples, only probe sets with 30 or higher signal intensity in at least one sample were used.

3. To use probe sets corresponding to known exon sequence, only probe sets with Evidence Level "Core" were used.

4. To avoid the influence of alternative splicing and non-specific hybridization, 5–8 were performed for probe sets of the Transcript Cluster with 8 or more probe sets for which conditions 1–3 were met.

5. To compare expression levels among probe sets in each sample, the rank of each probe set of the sample was decided based on the signal intensity.

6. One transcript cluster with probe sets for which conditions 1–3 were met were separated into 5′ and 3′ terminal groups at all possible cut off points so that each terminal group contains 4 or more probe set. ("cut off point" is only used in our algorithm to divide genome region into 5′ or 3′ terminal groups) For each sample, the average rank of probe sets in 5′ and 3′ terminal groups were calculated, respectively.

7. To detect genes with a clear expression level change before and behind the cut off points, it is confirmed that the difference in the average ranks of 5′ and 3′ terminal groups was 70% or more of the number of samples.

8. To reduce the possibility of false positives by measurement errors, the cut off points were identified as breakpoints only when at least one of the standard deviations of probe set ranks in 5′ or 3′ terminal groups was 2.0 or lower. Transcript clusters with candidate breakpoints were identified as candidate genes.

Our program for detecting fusion genes was written in Fortran95. One more program for drawing exon expression pattern of samples and location of exon in the genome database, as shown in the figures in this paper, was written in statistical language of R. We used Windows PC for both programs as a platform. Any machines installed with the Fortran95 and R would be able to be used for our purpose. Our source program will be available on direct request to the corresponding author.

### Evaluation of candidate genes

To take transcript isoforms of candidate genes into consideration, the transcript isoform information registered in UCSC Genome Browser ( http://genome.ucsc.edu/cgi-bin/hgGateway) "UCSC Gene" and "Ensembl Gene Prediction" was used. When the exon/intron structure of the aberrant transcript predicted from the exon expression profile of the candidate gene was similar to the registered transcript isoform, the gene was excluded from candidate genes. When the candidate gene (Transcript Cluster) corresponds to two or more RefSeq genes in UCSC Genome Browser, the gene was also excluded from candidate genes. When the exon expression profile of the screened sample in candidate genes was similar to the profile of the reference sample, the gene was excluded from candidate genes. Moreover, exon expression profiles of the candidate genes were evaluated by visual inspection in detail.

### TAIL-PCR, RT-PCR and one step RT-PCR

TAIL-PCR (thermal asymmetric interlaced-PCR) was performed with a slight modification of the original Yao-Guang Liu and Yuanling Chen’s high-efficiency TAIL-PCR protocol [18] for the identification of fusion counterpart. The primers and thermal cycling condition are shown in Tables 1, 2, and 3. For RT-PCR, TaKaRa Ex Taq Hot Start Version and 2 μl synthesized cDNA as template were used. Thermal cycling was carried out under the following conditions: 1 min at 95°C followed by 35 cycles of 15 sec at 95°C, 30 sec at 65°C, 2 min at 72°C. The primer pairs used in this experiment were designed to make the amplification product including the breakpoints of the fusion genes. For One Step RT-PCR, TaKaRa One Step SYBR PrimeScript RT-PCR Kit II was used according to the manufacturer’s instructions. 1 ng of total RNA from the dissected tumor cells was used as a template in each 20 μl reaction. Thermal cycling was carried out under the following conditions: 30 min at 50°C, 2 min at 94°C followed by 35 cycles of 30 sec at 94°C, 30 sec at 65°C, 1 min at 72°C. The primers for RT-PCR and One step RT-PCR are shown in Table 4.

**Table 1:** Gene-specific primers for TAIL-PCR

**Primer name**	**Sequence (5′-3′)**
ABCC4-TAIL0	CTGGTGGTGGGCGTTTCTGATATTCCC
ABCC4-TAIL1	ACGATGGACTCCAGTCCGGCCTTTGTCGAACACAC CACTGAAACAT
ABCC4-TAI L2	CCAGGCGCTTCACATCTCTTGACGTTTCC
ATP6V0A4-TAIL0	TTCCATGTGCCGCTGAACATGGGTTGG
ATP6V0A4-TAIL1	ACGATGGACTCCAGTCCGGCCAAAGATGTTCAAGGACTTGGAGAAGCAG
ATP6VOA4-TAI2	CTGGG1TfATCTCCCGGTAGCTGCCGAC
CDCA2-TAIL0	GCATTGCAGTTTTCCTTCTGCAGCTCC
CDCA2-TAIL1	ACGATGGACTCCAGTCCGGCCTGCTGCAGGGTCAGAGCAGGTTTG
CDCA2-TAIL2	CTTGATG CATATGCAAATCTGGGTCATGACG C
CEP250-TAIL0	GAGCTGGGTCTGTAGTATCCCAGTGG
CEP250-TAIL1	ACGATGGACTCCAGTCCGGCCTCAGTCGTTCCAGTTGTTGGCTG
CEP250-TAIL2	AGCAGTGTCTCCAGGAGGGATACTCTC
MACF1-TAIL0	CGATCATCTAGGAGCCGCTGGAGC
MACF1-TAIL1	ACGATGGACTCCAGTCCGGCCAACCAGCTGAGCAATGGCTCC
MACF1-TAIL2	CCCACAATGCAACAAAGCTTCCTGTAGCTG
RLF-TAIL0	CCATTCCTTCAGTCTCTACAGGAGTCAC
RLF-TAIL1	ACGATGGACTCCAGTCCGGCCAAGGAAGGGGTGTGGAAAAACCCAG
RLF-TAIL2	CTGTCTCAACAGCCAGTAGAAACGGAGG
SLCO4A1-TAIL0	CAGGAGCCCCATGATGAGTATGTAG
SLCO4A1-TAIL1	ACGATGGACTCCAGTCCGGCCACAGCAGACAGGCCTTGTCGATC
SLCO4A1-TAIL2	GCATTTCCCTGCAGTGGCATGGCC

**Table 2:** LAD primers and AC1 primer for TAIL-PCR

**Primer name**	**Sequence (5′—3′)**
LDA1	ACGATGGACTCCAGAGCGGCCGC(G/C/A)N(G/C/A)NNNGGAA
LDA2	ACGATGGACTCCAGAGCGGCCGC(G/C/T/)N(G/C/T)NNNGGTT
LDA3	ACGATGGACTCCAGAGCGGCCGC(G/C/A)(G/C/A)N(G/C/A)NNNCCAA
LDA4	ACGATGGACTCCAGAGCGGCCGC(G/C/T)(G/A/T)N(G/C/T/)NNNCGGT
LDA5	ACGATGGACTCCAGAGAG(A/T)GNAG(A/T)ANCA(A/T)AGG
LDA6	ACGATGGACTCCAGAG(A/T)GTGNAG(A/T)ANCANAGA
AC1	ACGATGGACTCCAGAG

**Table 3:** Thermal conditions for TAIL-PCR

**Pre-amplification**			**Primary TAIL-PCR**			**Secondary TAIL-PCR**		
**Step**	**Temperature (°C)**	**Time (min:sec)**	**Step**	**Temperature (°C)**	**Time (min:sec)**	**Step**	**Temperature (°C)**	**Time (min:sec)**
1	93	2:00	1	94	0:20	1	94	0:20
2	95	1:00	2	65	1:00	2	68	1:00
3	94	0:30	3	72	3:00	3	72	3:00
4	25	2:00	4	To step 1	1 time	4	94	0:20
5	Ramping to 72	0.5°C/s	5	94	0:20	5	68	1:00
6	72	3:00	6	68	1:00	6	72	3:00
7	94	0:30	7	72	3:00	7	94	0:20
8	60	1:00	8	94	0:20	8	50	1:00
9	72	3:00	9	68	1:00	9	72	3:00
10	Go to step7	10 times	10	72	3:00	10	To step 1	7 times
11	94	0:30	11	94	0:20	11	72	5:00
12	25	2:00	12	50	1:00			
13	Ramping to 72	0.5°C/s	13	72	3:00			
14	72	3:00	14	To step 5	13 times			
15	94	0:20	15	72	5:00			
16	58	1:00						
17	72	3:00						
18	Go to step 15	25 times						
19	72	5:00						

**Table 4:** Primers for RT-PCR

**Target fusion gene**	**Primer name**	**Orientation**	**Sequence (5′- 3′)**	**Amplicon size**
*DOCK5-CDCA2*	DOCK5-exonl	Forward	GAGGAGCTGTAGCAGCCTTAGTCG	371 bp
	CDCA2-TAIL2	Reverse	CTTGATGCATATGCAAATCTGGGTCATGACGC	
*DOCK5-CDCA2*	DOCK5-exon1	Forward	GAGGAGCTGTAGCAGCCTTAGTCG	760 bp
	CDCA2-TAIL0	Reverse	GCATTGCAGTTTTCCTTCTGCAGCTCC	
*ZMYND8-CEP250*	ZMYND8-exon18	Forward	TACATCAGGAGGCMAGCGACA	513 bp
	CEP250-TAIL2	Reverse	GCAGTGTCTCCAGGAGGGATACTCTC	
*ZMYND8-CEP250*	ZMYND8-exonl5	Forward	GCCGCTTTTACCGAAGGAGACT	1476 bp
	CE P250-exon27	Reverse	GCTGCTGCTCCGTGATATGAGA	
*RLF-ZMPSTE24*	RLF-TAIL2	Forward	CCCCCAGGCTACTGCTTTATCAAAACTA	445 bp
	ZMPSTE24-exon3	Reverse	CATAACCACAGAACCGTCCAGAAAG	
*RLF-ZMPSTE24*	RLF-exon1	Forward	GTTGCCTACGCGCTGGTG	2167 bp
	ZMPSTE24-exon10	Reverse	GATGTCCAGGATCTGTGACTGA	

The amplified PCR products were electrophoresed on 1.0% or 2.0% agarose gels, and were purified using GL Sciences MonoFas DNA purification kit I (GL Sciences, Tokyo, Japan). The purified products were sequenced using Applied Biosystems BigDye Terminator v3.1 Cycle Sequencing Kit (Life Technologies, Carlsbad, California), and the reaction products were purified using Promega Wizard MagneSil Sequencing Reaction Clean-Up System (Promega, Madison, WI). The purified samples were analyzed using Applied Biosystems 3130χ Genetic Analyzer.

## Results

### Development of fusion gene screening program

**Figure 1:**
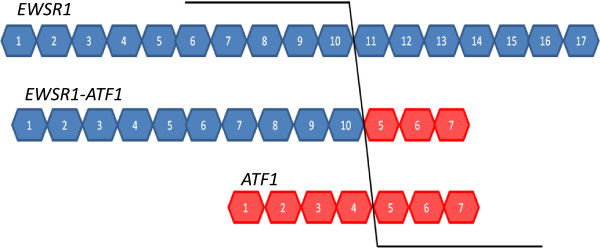
**Schema of EWSR1-ATF1 mRNA.** EWSR1 exon 1-10 fuse to ATF1 exon 5-7 by in-frame. Boxes with numbers represent the exon regions of the genes.

**Figure 2:**
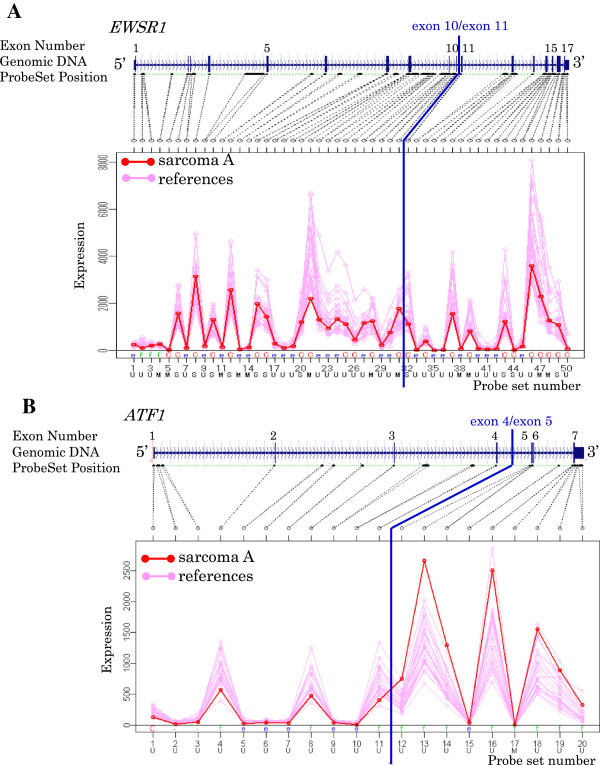
**Exon Array data of fusion partner genes.** Exon expression profiles of fusion partner genes EWSR1 **(A)** and ATF1 **(B)** are shown by line graphs with target areas of probe sets and genomic DNA structures. SarcomaA cell line and reference samples (breast cancer cell lines) are indicated by red and pink lines, respectively. Known breakpoints are shown by blue lines. Characters at top and bottom of probe set numbers indicate annotations: C = core, e = extended, f = full, U = unique, S = similar, M = mixed.

To profile the exon expression in fusion genes, SarcomaA which harbors the fusion gene *EWSR1-ATF1*, was used for Exon Array experiments (Figure 1). Exon expression profiles of *EWSR1* and *ATF1* were characterized (Figure 2), and the following features were observed. 1: Probe sets in the exon region had high signal intensity, and probe sets in the intron region had low signal intensity. 2: In some probe sets, all samples had equivalent signal intensity. In other probe sets, all samples had extremely low equivalence. 3: The expression signals vary in each probe set on a gene of one sample. 4: SarcomaA showed a change in the expression level at the breakpoint in comparison with breast cancer cell lines.

Then the fusion gene screening program was developed to detect fusion genes with an exon expression profile similar to that of *EWSR1* and *ATF1*.

**Figure 3:**
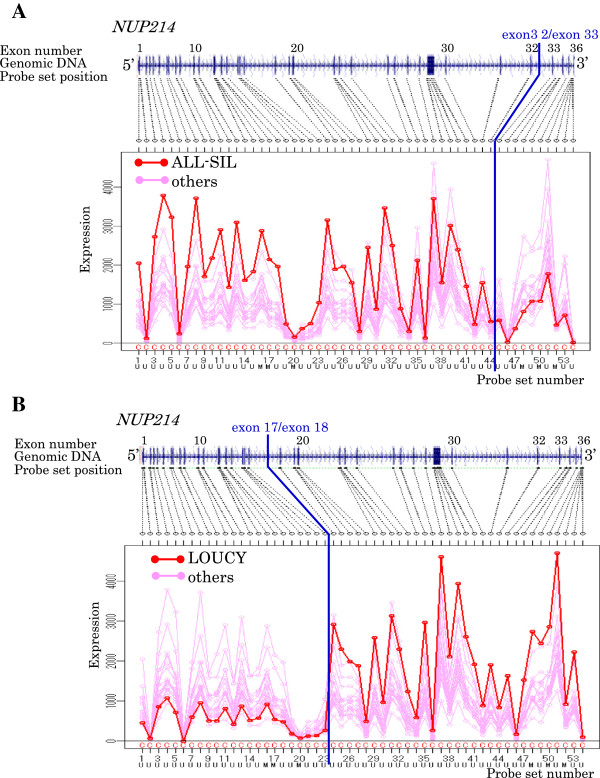
**Expression profiles of NUP214. Exon expression profiles of NUP214 (ENST00000359428) are shown by line graphs with target areas of probe sets and genomic DNA structures.** Examined cell line, **(A)**: ALL-SIL **(B)**: LOUCY, and reference samples (16 T-ALL cell lines) are indicated by red and pink lines, respectively. Predicted breakpoints are shown by blue lines. Characters at top and bottom of probe set numbers indicate annotations: C = core, U = unique, S = similar, M = mixed.

The detection performance of the developed program was examined using the Exon Array data of the T-ALL cell lines. The program selected the candidate genes: one gene in ALL-SIL, one gene in BE13, and two genes in LOUCY. NUP214, the partner gene of the known fusion genes, was detected in ALL-SIL and LOUCY. Other known fusion partner genes, ABL1 in ALL-SIL, NUP214 and ABL1 in BE13, SET in LOUCY, were not detected in this case, because the probe sets that could be used in the 5′ or 3′ terminal groups were three or less. Although the NUP214 gene was detected as a candidate gene in ALL-SIL and LOUCY, its exon expression profile was different between the two cell lines. While the expression decreases from the 5′ terminal side to the 3′ terminal side at the breakpoint in ALL-SIL, it was opposite in LOUCY. Thus it was confirmed that gene detection by the program did not depend on the direction of the expression change. Although breakpoints were detected at a different position in ALL-SIL and LOUCY, they corresponded to the position of reported breakpoints. It was confirmed that the breakpoint was detected accurately by the program (Figure 3).

### Candidate genes in breast and pancreatic cancer cell lines

**Figure 4:**
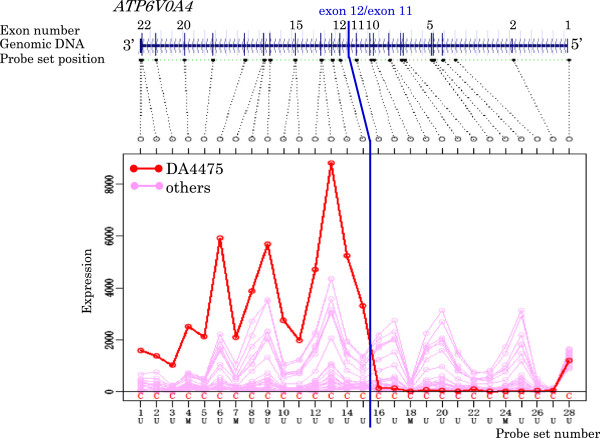
Exon expression profiles of candidate gene ATP6V0A4 in DU4475 cell.

**Figure 5:**
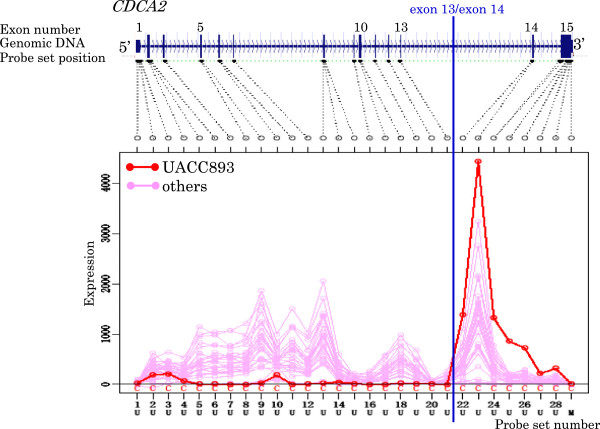
Exon expression profiles of candidate gene CDCA2 in UACC893 cell.

**Figure 6:**
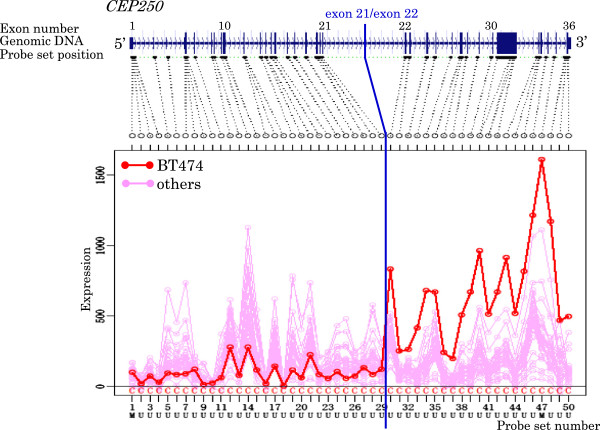
Exon expression profiles of candidate gene CEP250 in BT474 cell.

**Figure 7:**
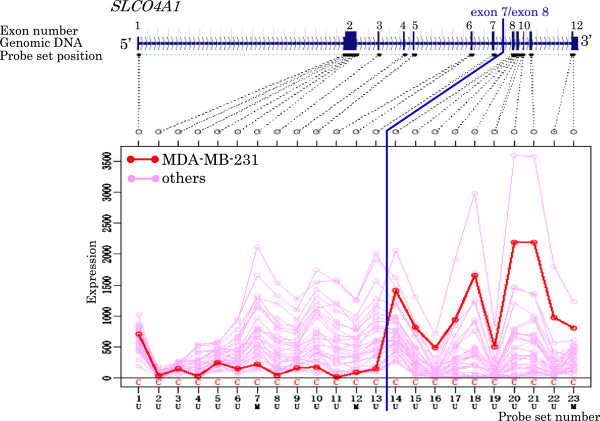
Exon expression profiles of candidate gene SLCO4A1 in MDA-MB-231 cell.

**Figure 8:**
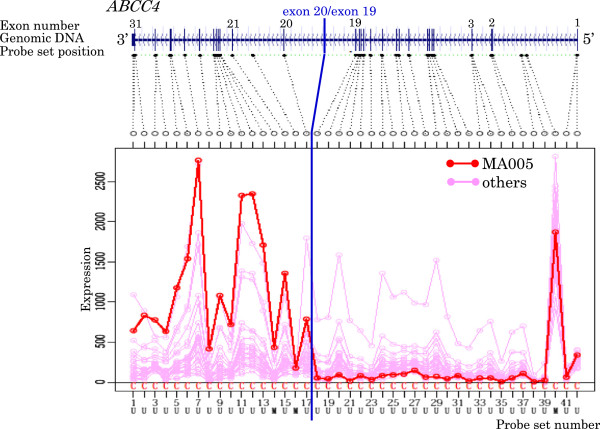
Exon expression profiles of candidate gene ABCC4 in MA005 cell.

**Figure 9:**
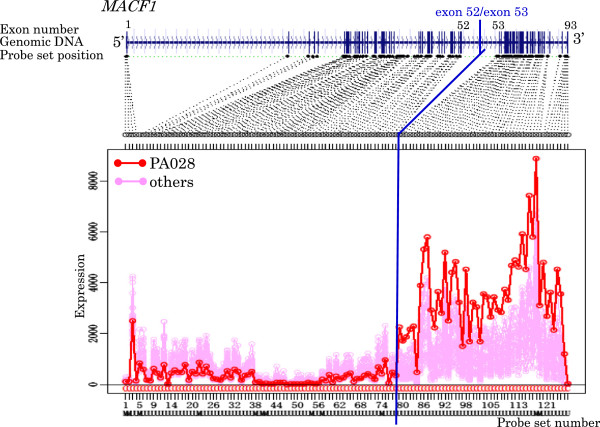
Exon expression profiles of candidate gene MACF1 in PA028 cell.

**Figure 10:**
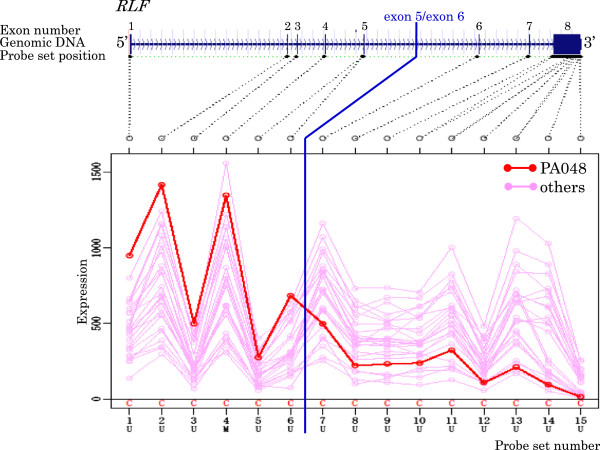
Exon expression profiles of candidate gene RLF in PA043 cell.

To discover the novel fusion gene in breast and pancreatic cancer cell lines, candidate genes were selected by the proposed methodology. As a result, 20 genes were selected in 24 breast cancer cell lines. Four of the selected genes were excluded from the candidates, because it was thought that the exon expression profiles of these 4 genes were influenced by known transcript isoforms. One gene was excluded, because a similar exon expression profile to the cancer cell line detected by the program was also observed in HMEC. As a result of the evaluation of the 15 remaining genes, 4 most attractive genes were selected as candidate genes in the breast cancer cell lines. In the 20 pancreatic cancer cell lines, 23 genes were selected by the program. Nine genes of them thought to be influenced by known transcript isoforms, and 3 genes that correspond to two or more RefSeq genes, respectively, were excluded from the candidate genes. As a result of evaluating the 11 remaining genes, the 3 most attractive genes were selected as candidate genes in the pancreatic cancer cell lines. Details are shown in Table 5 and Figures 3, 4, 5, 6, 7, 8, 9 and 10.

**Table 5:** Candidate genes

	**Transcript cluster ID**	**Gene symbol**	**Breakpoint**		**Examined sample**
			**Upstream probe set ID**	**Downstream probe set ID**	
Breast	3075381	*ATP6V0A4*	3075407	3075406	DU4475
	3090697	*cDcA2*	3090726	3090727	UACCS93
	3883309	*C’EP250*	3883348	3883349	BT474
	3892812	*SLCO4AJ*	3892835	3892837	MDA-MB-231
Pancreas	3521174	*ABCC4*	3521233	3521248	MA005
	2331505	*MACF1*	2331398	2331419	MA028
	2331771	*RLF*	2331793	2331801	PA043

Exon expression profiles of all selected gene by the program are shown in Additional file 1 and Additional file 2.

### Identification of novel fusion gene

It was attempted to identify unknown counterpart genes using TAIL-PCR from higher expression ends of selected candidate genes. In this research we did not carry out it from lower ends. TAIL-PCR is one of the methods by which an unknown sequence adjacent to an already-known sequence can be efficiently amplified [19]. As a result of fusion gene identification experiments for the 7 candidate genes, gene fusion fragments were acquired for 3 candidate genes. Additionally, the frequency of fusion genes evaluated in cell lines and clinical tissue samples using RT-PCR and One Step RT-PCR.

#### DOCK5-CDCA2

**Figure 11:**
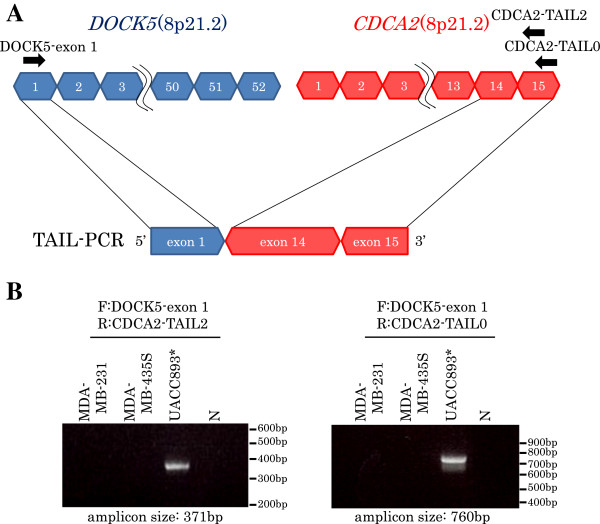
**TAIL-PCR detection and RT-PCR confirmation of fusion gene DOCK5-CDCA2.** Acquired fusion fragments by TAIL-PCR and exon structures of fusion partners are shown in **(A)** Red blocks are exons of candidate gene, blue blocks are exons of detected genes by TAIL-PCR. Arrows are primers for RT-PCR. RT-PCR confirmations for indicated samples are shown in **(B)** F: forward primer, R: reverse primer, *: detected samples in the program.

The upstream sequence of exon 14 of *CDCA2* gene (ENST00000380665) was acquired in breast cancer cell line UACC893. This sequence was part of the exon 1 of *DOCK5* gene (ENST00000276440) (Figure 11A). In addition, the fusion of *DOCK 5* exon 1 and *CDCA2* exon 14 was confirmed by RT-PCR (Figure 11B). But *DOCK5-CDCA2* fusion mRNA was not detected by RT-PCR in 111 breast cancer clinical tissues.

#### ZMYND8-CEP250

**Figure 12:**
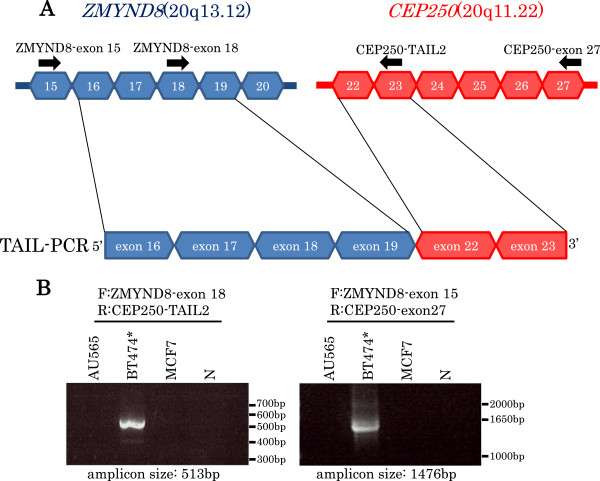
**TAIL-PCR detection and RT-PCR confirmation of fusion gene ZMYND8- CEP250.** Acquired fusion fragments by TAIL-PCR and exon structures of fusion partners are shown in **(A)** and **(B)** like Figure 11.

The upstream sequence of exon 22 of *CEP250* gene (ENST00000356095) was searched for in breast cancer cell line BT474, and was found to be a sequence from exon 16 to exon 19 of *ZMYND8* gene (ENST00000360911) (Figure 12A). The fusion of *ZMYND8* exon 19 and *CEP250* exon 22 was confirmed by RT-PCR (Figure 12B). But *ZMYND8-CEP250* fusion mRNA was not detected by RT-PCR in 111 breast cancer clinical tissues.

#### RLF-ZMPSTE24

**Figure 13:**
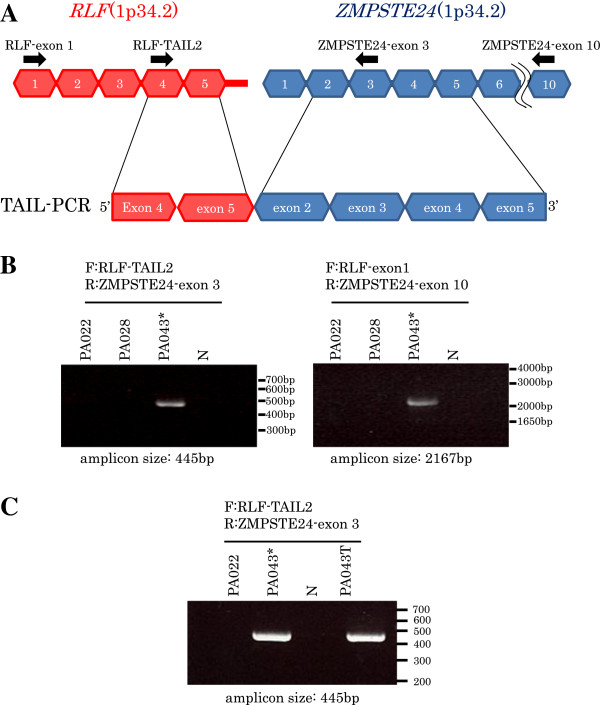
**TAIL-PCR detection and RT-PCR confirmation of fusion gene RLFZMPSTE24.** Acquired fusion fragments by TAIL-PCR and exon structures of fusion partners are shown in **(A)** and **(B)** like Figure 11, and RT-PCR detections for pancreatic cancer clinical tissue PA043T is shown in **(C)**.

The upstream sequence of exon 5 of *RLF* gene (ENST00000372771) was acquired in pancreatic cancer cell line PA043, and was found to be a sequence from exon 2 to part of exon 5 of *ZMPSTE24* gene (ENST00000372759) (Figure 13A). In addition, the fusion of *RLF* exon 5 and *ZMPSTE24* exon 2 was confirmed by RT-PCR (Figure 13B). *RLF-ZMPSTE24* fusion mRNA was detected by RT-PCR in pancreatic cancer clinical tissue, PA043T (Figure 13C). This tissue was the origin of the cell line PA043 where *RLF-ZMPSTE24* was first identified. The frequency of *RLF-ZMPSTE24* expression in pancreatic cancer patients was 1/58 (1.7%).

## Discussion

Here, a method is proposed to detect novel fusion genes using exon array data of tumor samples in combination with a new computational program.

### Development of new fusion gene detection program

This computational program is based on the following ideas.

#### Selection of probe set

Although a large number of probe sets are designed on Exon Array, it is known that there are some non-functional probes. Technical anomalies may give a false signal for un-functional probe sets due to cross-hybridization, saturation or an inherently weak and non-linear response. Actually, some probe sets for *EWSR1* and *ATF1* were thought to be un-functional probes. To minimize the effect of a false signal, non-functional probes were removed in step 1, 2, and 3 of the computational program.

#### Comparison of expression on different probe sets

Chromosome rearrangements often lead to the altered expression of 5′ or 3′ terminal regions of fusion partner genes by exchange of the transcriptional regulatory elements. The detection of sudden changes in the expression level between neighboring probe sets led to the discovery of breakpoints of fusion genes; however, the signal intensities obtained from different probes cannot be compared directly. Amplification and labeling efficiency are different in each RNA region. The hybridization property of probe sets on the array is also different in each probe set. Because of these biases, the signal intensity and dynamic range differ greatly between probe sets. Each probe set in the same gene has markedly different signal intensity; therefore, a normalizing method is needed to compare the signal intensities generated from different probe sets. On the other hand, signal intensities from different samples on the same probe sets can be compared because the biases are the same for all samples. In the program, samples were ranked using the signal intensities for each probe set in a gene. The change in rank of a sample implies intragenic exon expression change.

#### Grouping and average calculation of probe sets

Many genes have alternative transcript isoforms in vivo. Alternative splicing may contribute to expression differences between neighboring exons (probe sets), leading to a rank change. Moreover, because hybridization reactions on a great number of probes were performed under only one experimental condition in microarray experiments, non-specific cross hybridization cannot be avoided completely. The generated non-specific signals may influence the rank. Thus, rank changes between neighboring probe sets are thought to be observed frequently, and make it difficult to find the breakpoint. In the developed program, probe sets in the gene were divided into 5′ and 3′ terminal groups, and the average ranks of the probe set in each group were compared. The influences of unexpected rank changes were mitigated by this process.

#### Exclusion of false positives because of quantitative determination error margin

When the gene expression level is similar between samples, rank changes might take place at random due to quantitative determination error margins in Exon Array data, influencing the detection of breakpoints. False detection was decreased by monitoring the decentralization of a sample’s rank.

The main feature of the program is that expression levels between probe sets can be compared by replacing the expression signal intensity with the rank. In general, expression levels were not compared between probe sets in gene or exon expression analysis by microarray. In this research, the developed program and evaluation of candidates chose seven candidate genes, and three novel fusion genes were identified by TAIL-PCR and RT-PCR; therefore, it is thought that the proposed method is very efficient for fusion gene discovery.

There existed fusion gene detection methods through transcript analysis by microarrays before. However, these methods were restrictive ones for confirmation of known fusion gene or for detecting some known partner genes [20-23].

The detection method for novel fusion genes using Exon Array has been reported by Eva Lin, in addition to this research [24]. Lin et al. detected intragenic expression changes of the *ALK* gene in lung, breast, and colon cancer. Based on their results, fusion gene *EML4-ALK* was identified using 5′RACE (rapid amplification cDNA end). Although fusion gene *EML4-ALK* was originally discovered in lung cancer, it had not been discovered in other cancers before their study. Their methods also detect the expression level change between 5′ and 3′ terminal groups of a gene for fusion gene discovery as well as this report. To compare the expression level between probe sets, they developed the following method. First, the mean value and standard deviation of the signal value of each probe set were calculated for all samples. Signal intensity was then standardized by subtracting its mean and dividing by its standard deviation. The standardized value was used as an index of the expression level of each probe set. The probe sets were then separated in a transcript cluster into 5′ and 3′ terminal groups by one arbitrary point, and the expression level change was monitored between groups by t-test.

Comparing the proposed methodology with Lin’s method, a common feature is that signal intensity is normalized based on the relative relation to reference samples, aiming to compare the expression levels of all probe sets in a gene. The most important difference is the strategy of normalizing. In Lin’s method, it is thought that normalized values have a fixed quantity, which is an advantage to evaluate whether the magnitude of the change is significant; however, this is influenced easily by outlier intensities, which are generated frequently in microarray experiments. On the other hand, in the developed program, the magnitude of the change is not evaluated appropriately, but it has the advantage that the result is not influenced easily by the outlier value because the expression intensity is converted into the rank.

### Points to be improved and limitations

The analysis result would possibly change depending on the selection of reference samples, because signal intensities are converted into relative values by comparing with other samples. Lin’s method has the same problem. It is thought that ideal reference samples for the program would show moderate variance of the gene expression level. Although cancer cell lines and healthy cells from the same organ were used in this research, further examination is necessary to assess whether this is the best choice. In addition, parameter optimization (degree of rank change, standard deviation and so on) for the reference samples is required.

The following points are limitations of this method, and alternative methods are needed. As this method detects the intragenic expression change in fusion partner genes, the method cannot detect the genes with no significant expression change between exons. Additionally, breakpoint detection from exon array data depends on the genomic position of the probe set. Thus, this method is not able to identify breakpoints on genomic DNA in detail.

### Contribution of the fusion genes to cancer

The discovery of fusion genes that contribute to the pathology (tumorigenesis, metastasis etc.) are hoped from the viewpoint of the diagnosis and treatment of cancer. Considering the functional aspect of the fusion gene, it is important to incorporate other information, such as protein domain composition, when prioritizing novel, biologically relevant genomic aberrations [25].

Although three novel fusion genes were identified in this research, their function and contribution to cancer are unclear.

#### DOCK5-CDCA2

DOCK5 (dedicator of cytokinesis 5) is a member of the DOCK family of guanine nucleotide exchange factors which function as activators of small G proteins [26]. Although DOCK5 is predicted to activate the small G protein Rho and Rac, its function and signaling properties are poorly understood. CDCA2 (cell division cycle associated 2) recruits protein phosphatase 1 to mitotic chromatin at anaphase and into the following interphase, regulating the chromosome structure during mitosis [27]. Because *DOCK5* and *CDCA2* show out-of-frame fusion, it is thought that the amino acid sequence of *CDCA2* is disrupted and a premature termination codon appears in *CDCA2* exon 14. The fusion gene might therefore produce a short protein, 42aa (14aa from *DOCK5* exon 1, and 28aa from *CDCA2* exon 14). No functional protein domains have been found so the function of the fusion protein is unclear. Significant chromosome loss and underexpression of *DOCK5* have been reported in osteosarcoma [28]. DOCK5 dysfunction might contribute to tumors.

#### ZMYND8-CEP250

ZMYND8 is a member of RACK (receptor for activated C-kinase) family proteins that anchor activated protein kinase C (PKC). ZMYND8 interacts specifically with PKCβI and is predicted to regulate subcellular localization and activity [29]. In addition, ZMYND8 contains a bromo domain, a PWWP domain, and two zinc fingers, and is thought to be a transcriptional regulator. CEP250 is a core centrosomal protein required for centriole-centriole cohesion during interphase of the cell cycle [30], but details of the mechanism are not well known. *ZMYND8-CEP250* is also an out-of-frame fusion gene, so a premature termination codon appears in *CEP250* exon 24 and is likely to express a 1121aa protein (994aa from *ZMYND8* exon 1–19, and 127aa from *CEP250* exon 22–24). The down-regulation of PKCβ1 protein expression has been reported in colon cancer [31]. The PKCβ1 binding site in the C terminal region of ZMYND8 racks in the predicted fusion protein. Formation of the fusion gene may lead to the low activity of PKCB1, and may contribute to cancer, or deregulation of the transcript regulatory network managed by ZMYND8 might cause cancer.

#### RLF-ZMPSTE24

RLF is predicted as a transcription factor with zinc fingers from the amino acid sequence. It is reported that RLF forms a fusion gene with the LMYC gene in lung cancer [32]. The fusion gene RLF-LMYC contributes to carcinogenesis by changing the LMYC manifestation of a gene [33]. ZMPSTE24 performs a critical endoproteolytic cleavage step to generate mature lamin A, a major component of the nuclear lamina and nuclear skeleton [34]. Lack of functional ZMPSTE24 results in progeroid phenotypes, including genomic instability in mice and humans [35,36]. *RLF-ZMPSTE24* is an in-frame fusion gene, which may expresses the 704aa protein (270aa from *RLF* exon 1–5, and 434aa from *ZMPSTE24* exon 2–10). The known function domains of *RLF* are not contained in the fusion gene, and no change of *ZMPSTE24* expression level is observed in Exon Array data. Functional change of ZMPSTE24 may induce DNA damage and lead to cancer.

### Genomic structure of the fusion genes

*RLF* and *ZMPSTE24* genes located on chromosome 1, approximately 20 kb apart, have the same orientation. Southern blot analysis with a probe hybridizing to *RLF* intron 5 region showed chromosome rearrangement (data not shown), and a fragment that is part of *RLF* intron 5 fused to a part of *ZMPSTE24* intron 1 was obtained by TAIL-PCR for the upstream region of *ZMPSTE24* exon 2 on genomic DNA (data not shown). Both parts fused in the opposite orientation; therefore, the cause of the gene fusion, *RLF-ZMPSTE24*, might be chromosome inversion with some deletion. *ZMYND8* and *CEP250* genes were located on chromosome 20, approximately 12 Mb apart, in opposite orientation. *DOCK5* and *CDCA2* genes were located on chromosome 8, approximately 50Kb apart, in the same orientation. The mechanisms of gene fusions remain to be revealed.

The proposed method might be applied to not only Exon Array but also the Affymetrx GeneChip Gene 1.0 ST Array (Gene Array) with some improvements. Gene Array, in which each of the 28,869 genes is represented on the array by approximately 26 probes spread along the full length of the gene, is widely used for global gene expression analysis. Using this method for more samples, it is thought that fusion genes can be identified. This is expected to lead to new diagnostic methods and treatment strategies.

## Competing interests

The authors declare that they have no competing financial interests or other conflicts of interest.

## Authors’ contributions

YW carried out sample preparation, Exon Array Experiments, TAIL-PCR, manuscript writing and helped to develop the algorithms. MM developed the majority of algorithms, helped to draft the manuscript. MS contributed cell cultue and TAIL-PCR, helped to draft the manuscript. MU and SM contributed statistical support and data processing. KN contributed preparation of clinical samples. TN and YM contributed to study conception, and critical manuscript review. All authors read and approved the final manuscript.

## Supplementary Material

Additional file 1 Selected genes by the program in 24 breast cancer cell lines. Click here for file

Additional file 2 Selected genes by the program in 20 pancreatic cell lines. Click here for file
