# Supplementary material for: Development of detection method for novel fusion gene using GeneChip exon array
Source: J Clin Bioinforma. 2014 Feb 18;4:3. doi: 10.1186/2043-9113-4-3 (PMC3937068; doi:10.1186/2043-9113-4-3)
Supplement: Additional file 1 — Selected genes by the program in 24 breast cancer cell lines. [file 2043-9113-4-3-S1.pdf]

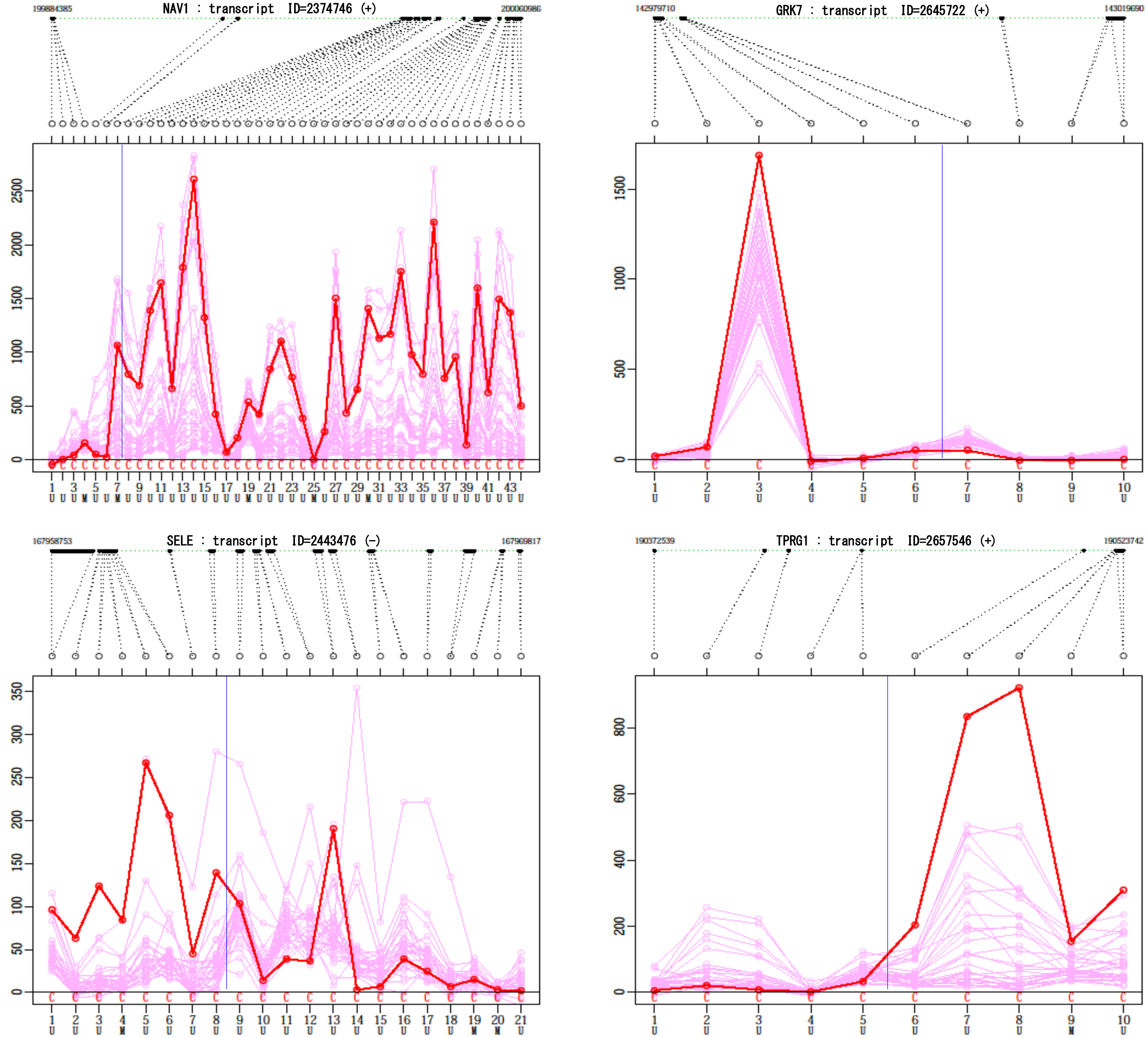

Additional file 1 -1/5

selected genes by the program in 24 breast cancer cell lines

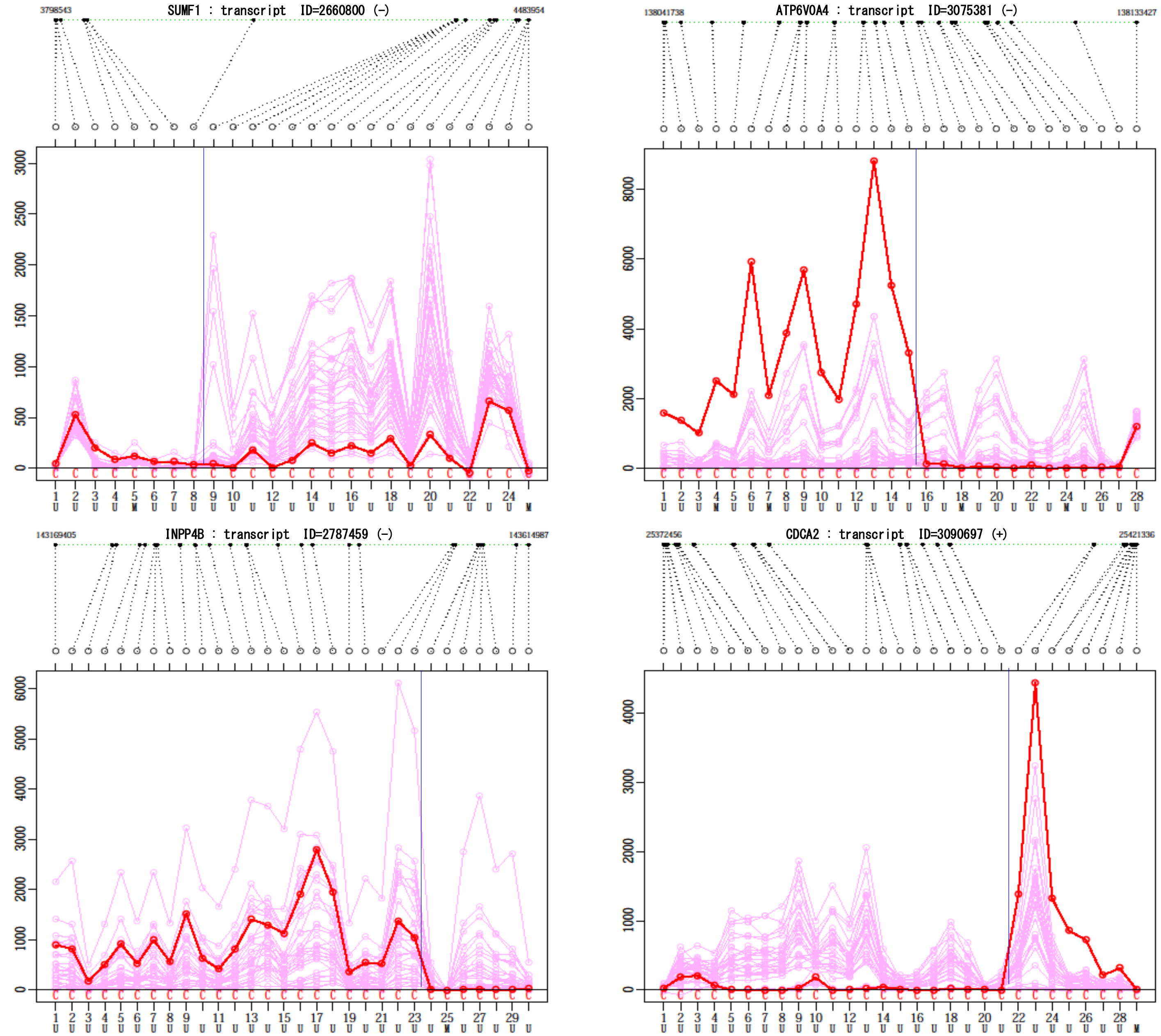

Additional file 1 -2/5

selected genes by the program in 24 breast cancer cell lines

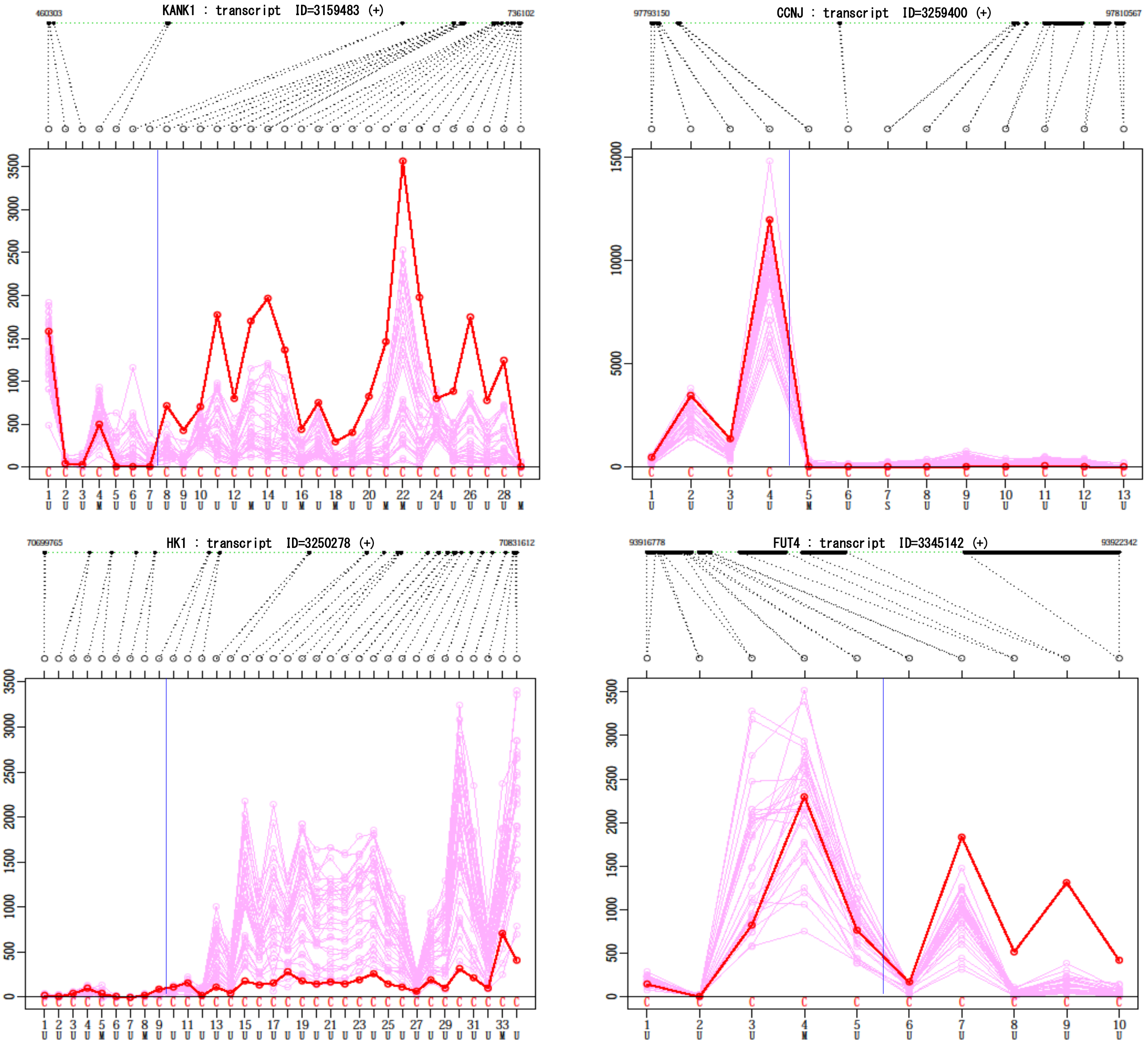

Additional file 1 -3/5

selected genes by the program in 24 breast cancer cell lines

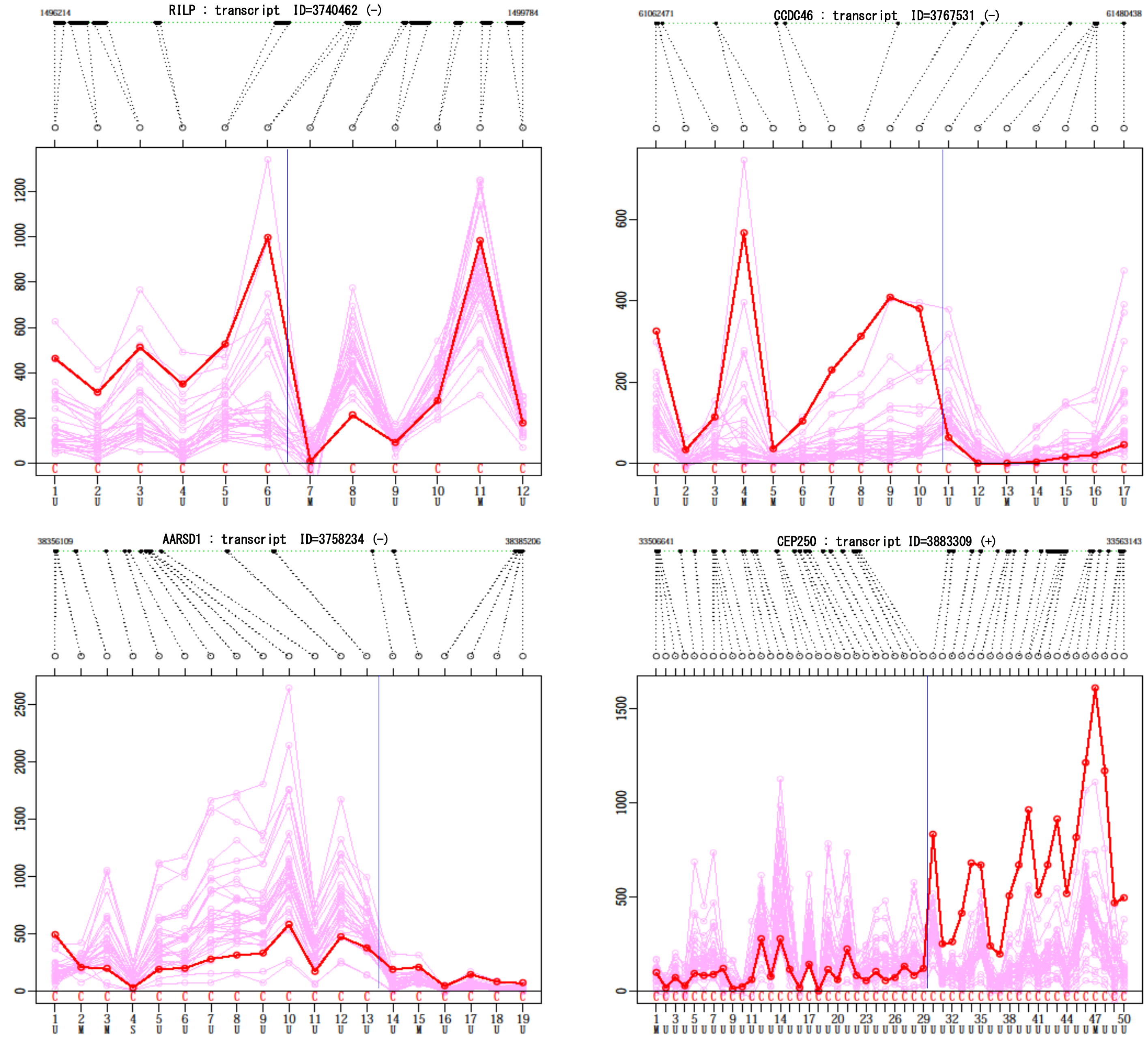

Additional file 1 -4/5

selected genes by the program in 24 breast cancer cell lines

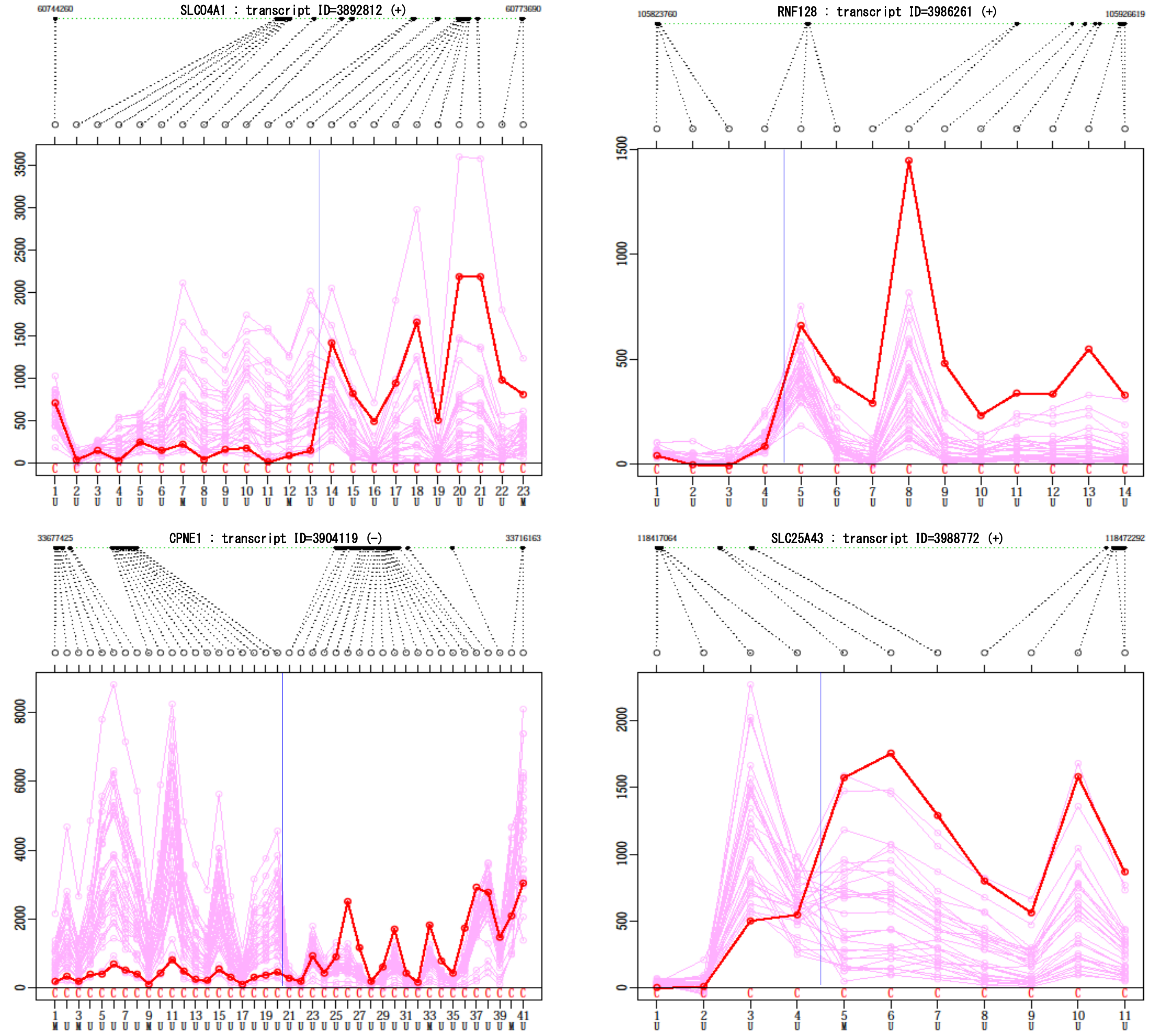

Additional file 1 -5/5

selected genes by the program in 24 breast cancer cell lines
