# Supplementary material for: Development of detection method for novel fusion gene using GeneChip exon array
Source: J Clin Bioinforma. 2014 Feb 18;4:3. doi: 10.1186/2043-9113-4-3 (PMC3937068; doi:10.1186/2043-9113-4-3)
Supplement: Additional file 2 — Selected genes by the program in 20 pancreatic cell lines. [file 2043-9113-4-3-S2.pdf]

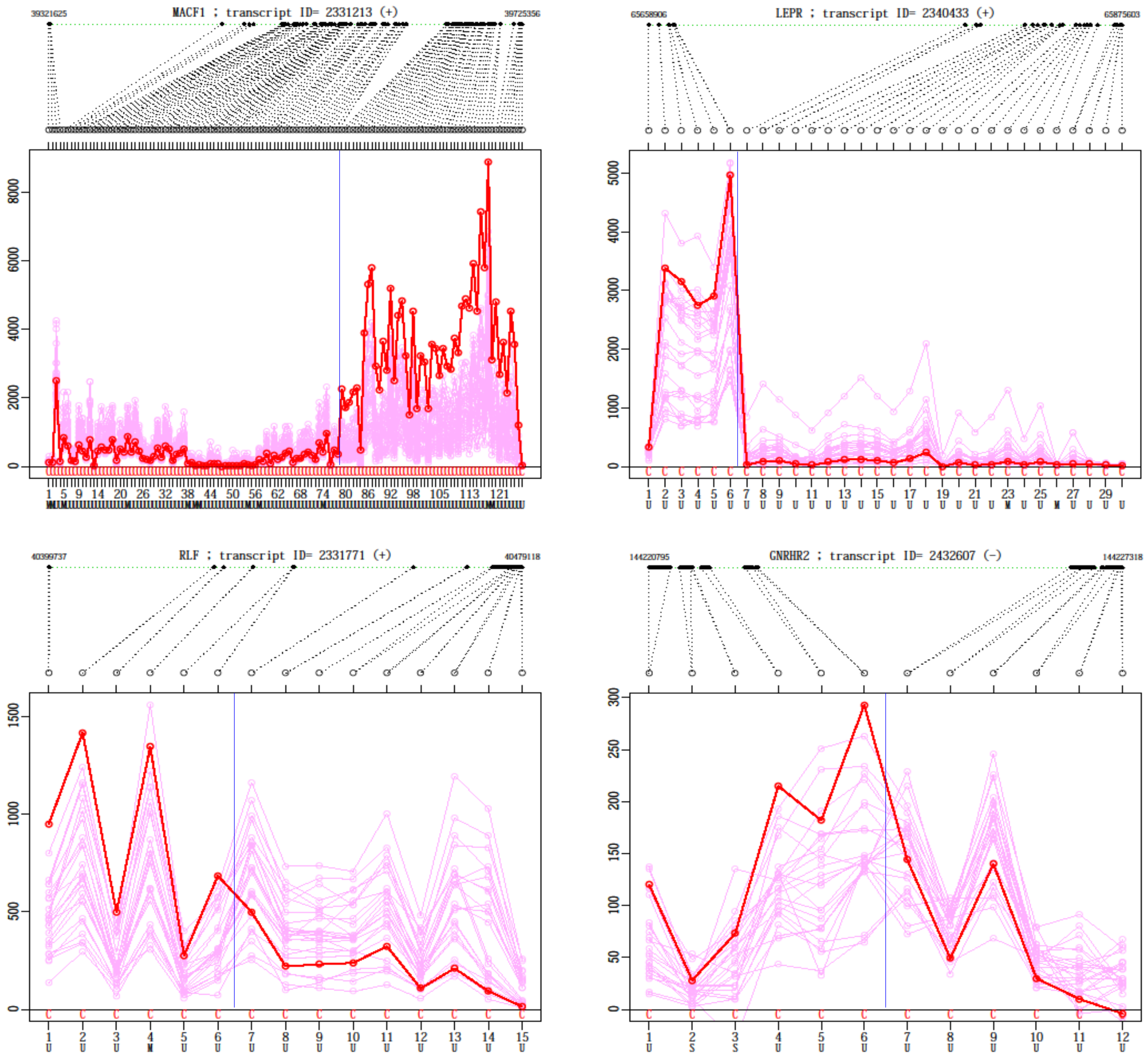

Additional file 2 -1/6

selected genes by the program in 20 pancreatic cell lines

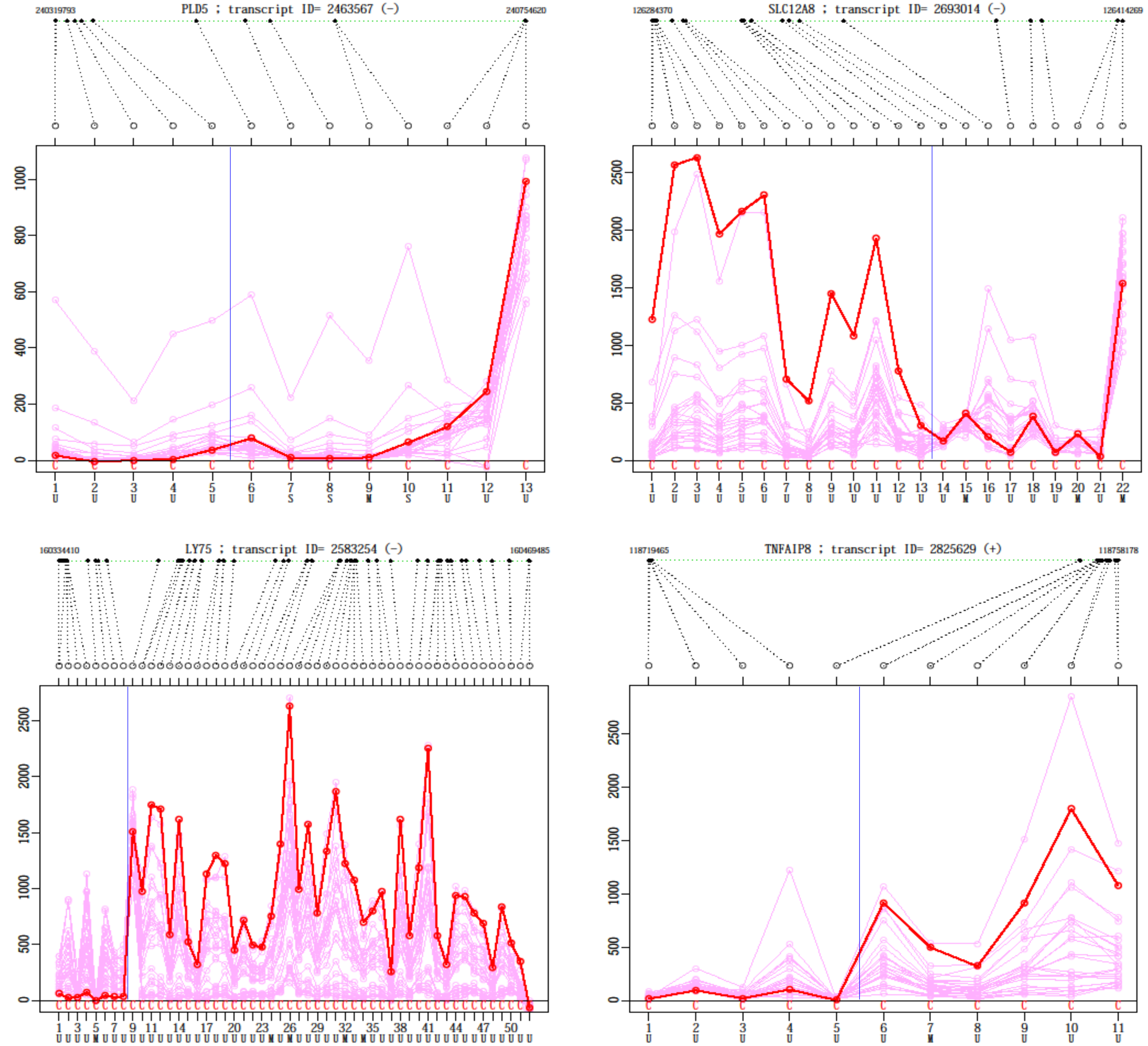

Additional file 2 -2/6

selected genes by the program in 20 pancreatic cell lines

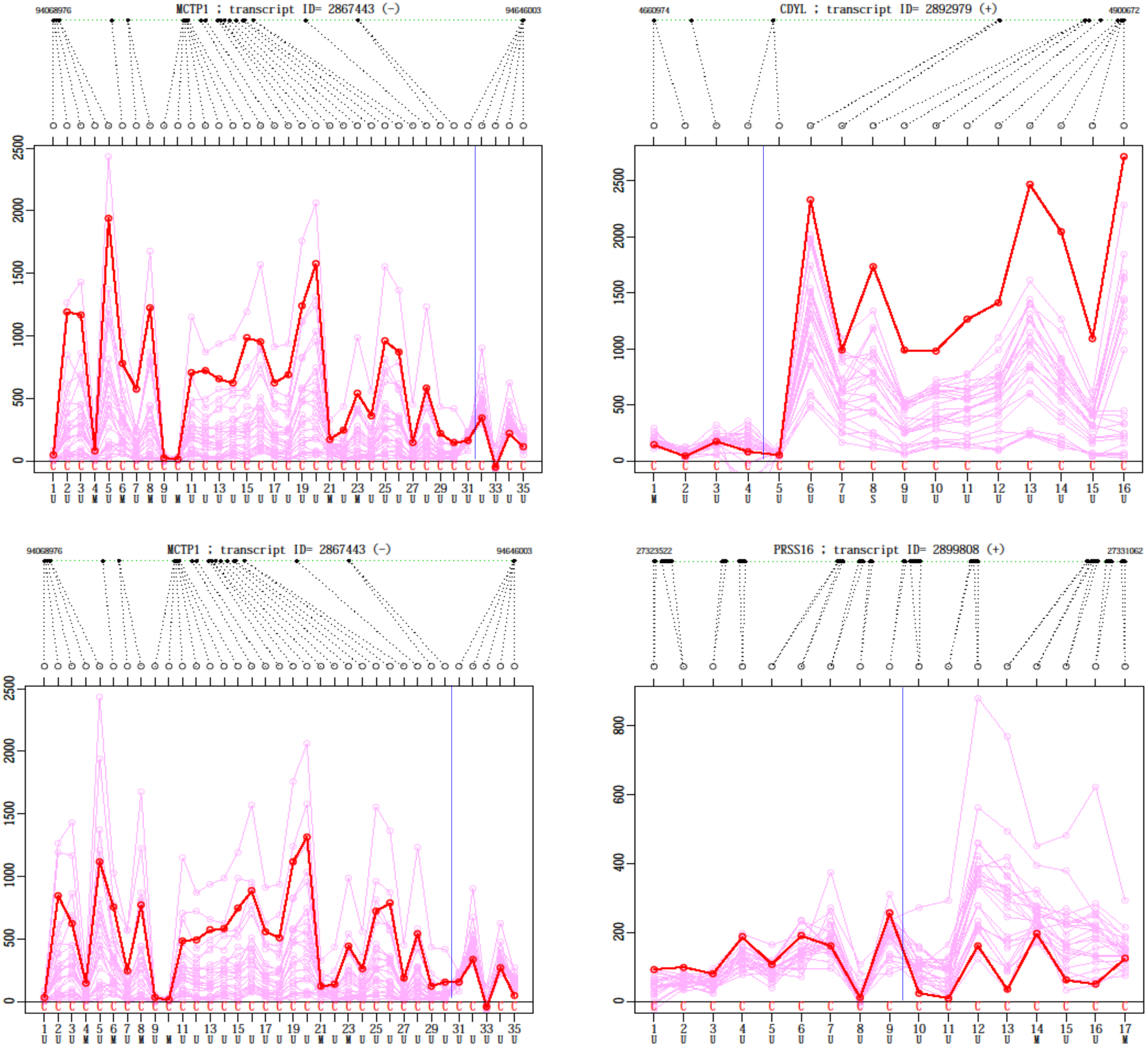

Additional file 2 -3/6

selected genes by the program in 20 pancreatic cell lines

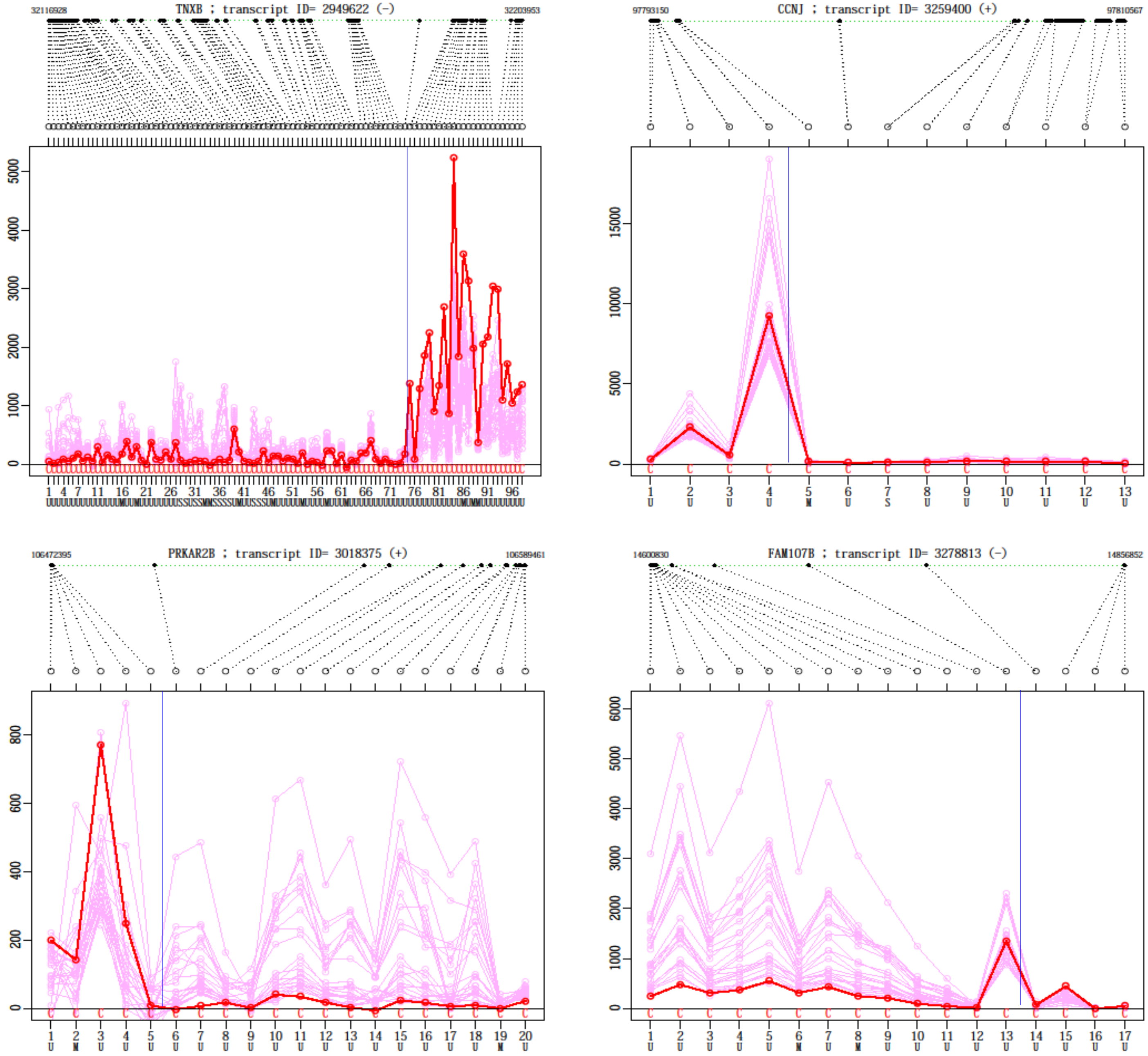

Additional file 2 -4/6

selected genes by the program in 20 pancreatic cell lines

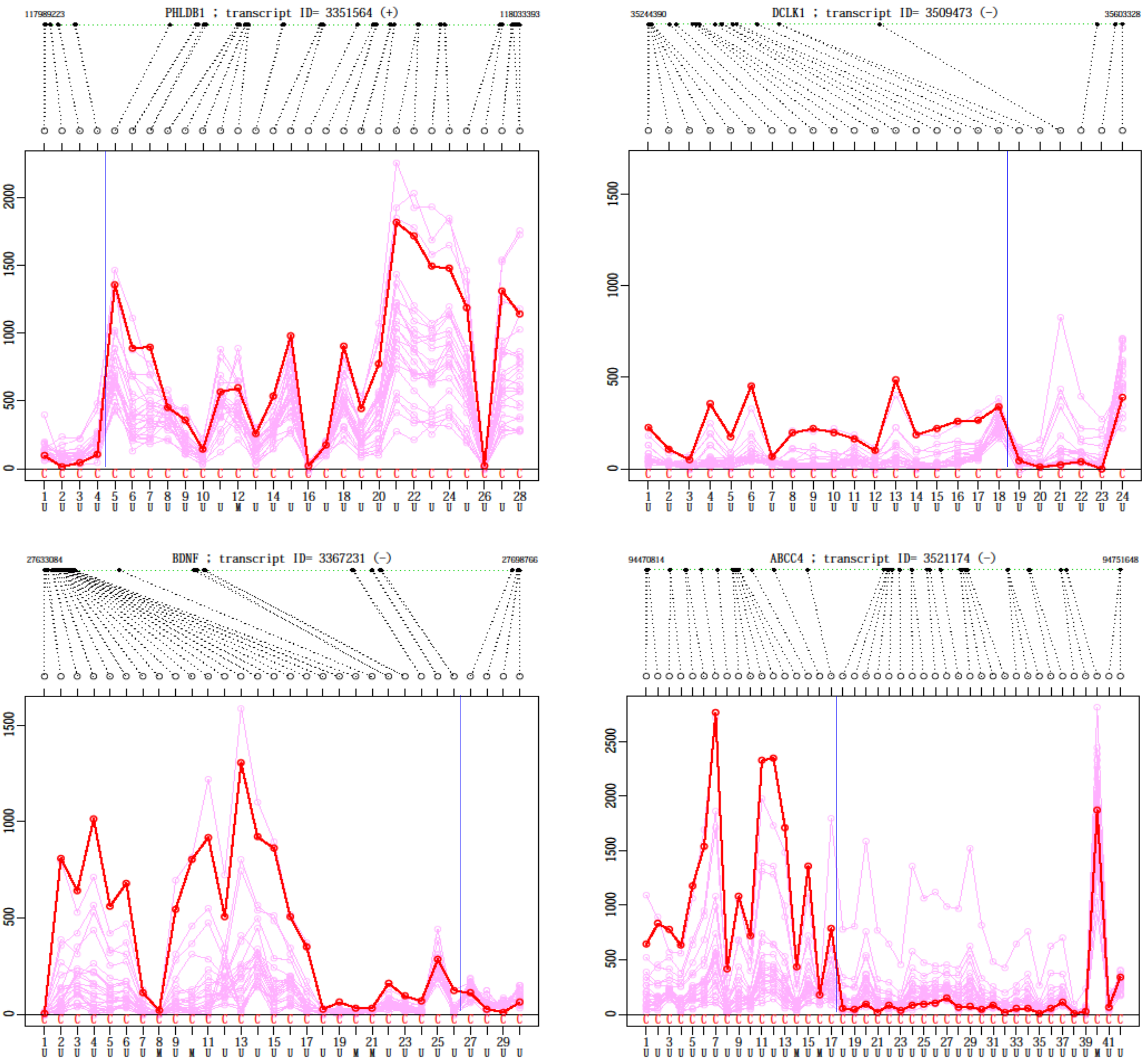

Additional file 2 -5/6

selected genes by the program in 20 pancreatic cell lines

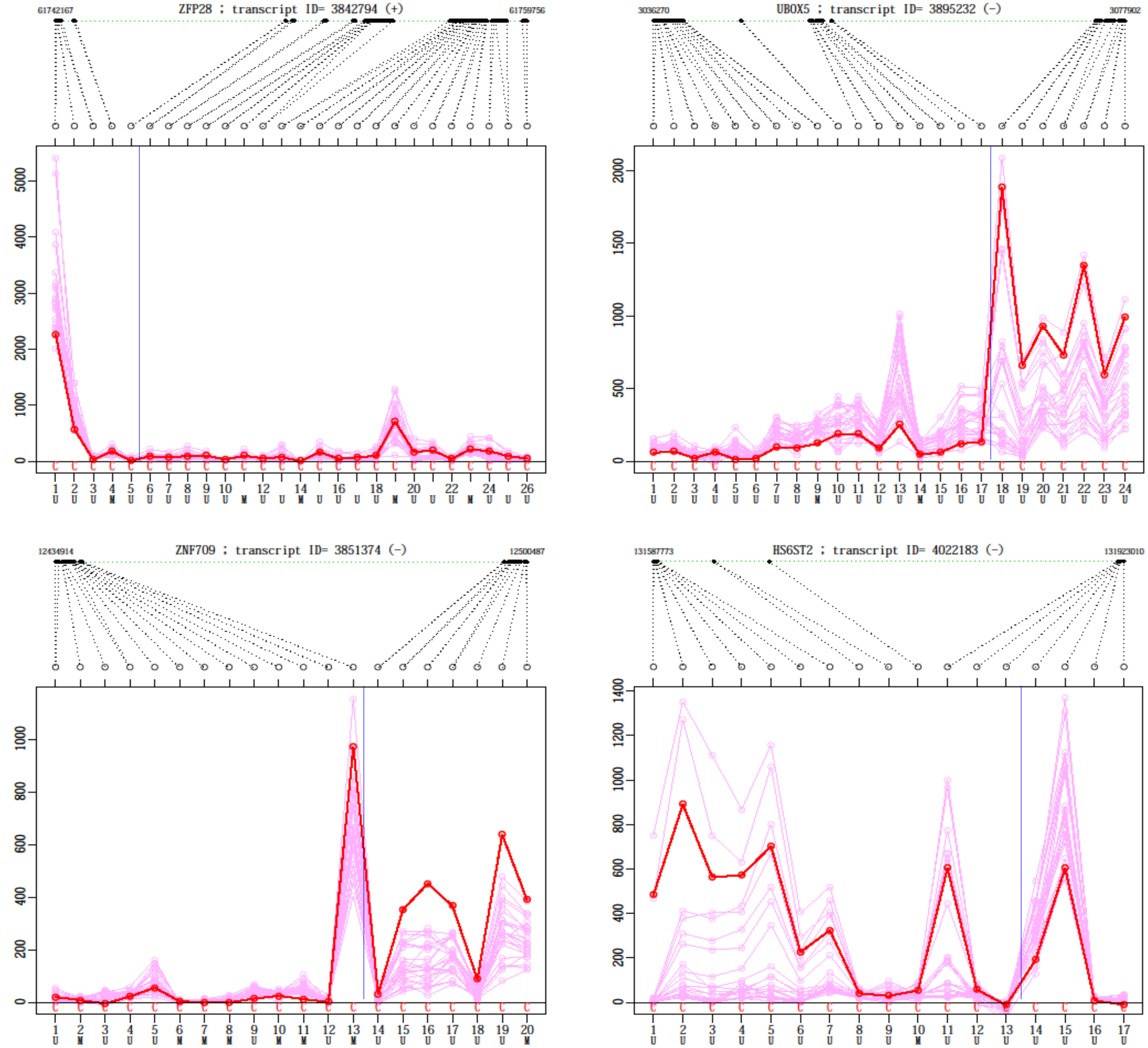

Additional file 2 -6/6

selected genes by the program in 20 pancreatic cell lines
